# Effect of methanol extract of Dicranopteris linearis against carbon tetrachloride- induced acute liver injury in rats

**DOI:** 10.1186/1472-6882-14-123

**Published:** 2014-04-04

**Authors:** Farah Hidayah Kamisan, Farhana Yahya, Siti Syariah Mamat, Mohamad Fauzi Fahmi Kamarolzaman, Norhafizah Mohtarrudin, Teh Lay Kek, Mohd Zaki Salleh, Mohd Khairi Hussain, Zainul Amiruddin Zakaria

**Affiliations:** 1 Department of Biomedical Science, Faculty of Medicine and Health Sciences, Universiti Putra Malaysia, 43400 UPM Serdang, Selangor, Malaysia; 2 Department of Pathology, Faculty of Medicine & Health Sciences, Universiti Putra Malaysia, 43400 UPM Serdang, Selangor, Malaysia; 3 Integrative Pharmacogenomics Institute (iPROMISE), Level 7, FF3, Universiti Teknologi MARA, Puncak Alam Campus, 42300 Puncak Alam, Selangor, Malaysia

**Keywords:** *Dicranopteris linearis*, Methanol extract, Carbon tetrachloride, Antioxidant, Hepatoprotective effect

## Abstract

**Background:**

*Dicranopteris linearis* (family Gleicheniaceae) has been reported to possess anti-inflammatory and antioxidant activities but no attempt has been made to study its hepatoprotective potential. The aim of the present study was to determine the hepatoprotective effect of methanol extracts of *D. linearis* (MEDL) against carbon tetrachloride (CCl_4_)-induced acute liver injury in rats.

**Methods:**

6 groups (n = 6) of rats received oral test solutions: 10% dimethyl sulfoxide (DMSO), 200 mg/kg silymarin, or MEDL (50, 250, and 500 mg/kg), once daily for 7 consecutive days, followed by hepatotoxicity induction with CCl_4_. Blood and liver were collected for biochemical and microscopic analysis. The extract was also subjected to antioxidant studies (e.g. 2, 2-diphenyl-1-picrylhydrazyl (DPPH)- and superoxide anion-radical scavenging assays, oxygen radical absorbance capacity (ORAC) test and total phenolic content (TPC) determination), phytochemical screening and HPLC analysis.

**Results:**

Pretreatment with MEDL and silymarin significantly (P < 0.05) reduced the serum levels of AST, ALT and ALP, which were increased significantly (P < 0.05) in DMSO-pretreated group following treatment with CCl_4_. Histological analysis of liver tissues in groups pretreated with MEDL and silymarin showed mild necrosis and inflammation of the hepatocytes compared to the DMSO-pretreated group (negative control group). The MEDL showed higher DPPH- and superoxide anion-radical scavenging activity as well as high TPC and ORAC values indicating high antioxidant activity.

**Conclusions:**

MEDL exerts hepatoprotective activity that could be partly contributed by its antioxidant activity and high phenolic content, and hence demands further investigation.

## Background

The liver has a pivotal role in regulating many important functions, such as metabolism, secretion, storage; and plays a role in regulating various physiological processes [1]. Furthermore, it is involved in detoxification of a variety of drugs and xenobiotics and thereforeat increased susceptible to the toxicity from these agents. It is frequently abused by poor drug habits and alcohol and widely exposed to environmental toxins, prescribed and over-the-counter drugs, which cause various liver diseases [2]. Liver diseases have become aworldwide problem as a result of extremely poor prognosis and high mortality due to the lack of effective prevention or drug available that stimulates liver function, offer protection against liver damage, or help to regenerate hepatic cells [3,4]. Therefore, attempts are perpetually being made to discover new treatment for liver disease, and the discovering process have been paid great attention to the investigation of the efficacy of plant-based drugs used in traditional medicine as they are cheap and have little side effects. Besides, WHO reported that 80% of the world population relies mainly on plant-based drugs [5].

In lieu of the aforementioned problem, patients suffering from liver diseases have turned to complementary and alternative medicine (CAM), which includes among others the use of herbal medicines as their sources of hepatoprotective agents. According to White et al. [6], over 50% of patients that required health care used CAM either in conjunction with, or separate from, conventional health care. Despite its uses for treatment of various types of diseases, the use of CAM is also popular in patients with liver disease but is not well documented [6]. According to Bhawna and Kumar [7], hepatoprotective plants contain a variety of phytochemicals like phenols, coumarins, monoterpenes, glycosides, alkaloids, and xanthenes. In addition, hepatoprotective properties are related with phytoextracts or phytocompounds rich in natural antioxidants as reported from previous studies [8-10]. One of the plants that have been studied extensively in our laboratory for its medicinal potentials is *Dicranopteris linearis* (L. (Gleicheniaceae), locally known as “resam”, is common in secondary forests and grows well in poor clay soil [11]. *D. linearis* has been used in Malay traditional medicine to reduce body temperature and to control fever [12]. In addition, there are few reports of its traditional uses in other parts of the world, with only 2 reports describing its use to treat external wounds, ulcers, and boils by the people of Papua New Guinea, to eliminate intestinal worms by the people of Indochina, and to treat asthma and female sterility by the tribes living on an Indian mountain [13]. Scientifically, the leaf extracts of *D. linearis* have been reported to possess antinociceptive, anti-inflammatory and antipyretic [12], gastroprotective [14], antistaphylococcal [15], antioxidant [16], and anticancer activities [17] properties.

The present study was performed based on three reasons, namely: i) the previous reports on the anti-inflammatory and antioxidant activities of *D. linearis* leaves; ii) the reports linking the anti-inflammatory and antioxidant activities to the hepatoprotective mechanism [3,18,19], and; iii) no scientific report to date to prove on the hepatoprotective potential of *D. linearis* leaves. It is postulated that *D. linearis* leaves will exert hepatoprotective activity that could be linked to its antioxidant activity. Therefore, the aim of the present study was to determine the hepatoprotective activity of methanol extract of *D. linearis* (MEDL) using the carbon tetrachloride (CCl_4_)-induced acute liver damage in rats model. In addition, the antioxidant activity, phytochemical content and HPLC profile of MEMM were also verified to support the hepatoprotective potential of MEDL. The hepatoprotective potential of the MEDL was compared with silymarin, a known, commercially available hepatoprotective agent.

## Methods

### Collection of plant material

The plant material (*D. linearis*) was obtained from its natural habitat in Serdang, Selangor, Malaysia. The plant was authenticated by Dr. Shamsul Khamis, a botanist at the Institute of Bioscience (IBS), Universiti Putra Malaysia (UPM), Serdang, Selangor, Malaysia, and voucher specimen SK 1987/11 was deposited in the herbarium of the IBS, UPM. The leaves were shade-dried for a week at room temperature (27 ± 2°C) and powdered mechanically.

### Preparation of plant extract

The powdered dried leaves were weighed (160 g) and then soaked (72 hours; room temperature) in methanol in the ratio of 1:20 (w/v). The solutions were collected and filtered with cotton wool followed by Whatman No. 1 filter paper. This procedure was repeated three times. Then, the collected supernatant was pooled together and evaporated using a rotary vacuum evaporator at 40°C under reduced pressure. The evaporation of the MEDL resulted in a yield of 48.4 g (30.3%). The crude dried extract obtained was kept at 4°C prior to use.

### Phytochemical screening

The phytochemical screening was carried out to determine the presence of alkaloids, saponins, flavonoids, hydrolysable and condensed tannins, triterpenes and steroids according to the method described by Ikhiri et al. [20]. Analyses were performed based on 5 g of dried powder material and 100 mg of extract (organic).

### HPLC analysis of MEDL

The HPLC analysis of MEDL was performed according to the previous report [21] but with slight modifications. Briefly, 10 mg of MEDL was dissolved in 1 ml methanol and then filtered through the membrane filter (pore size 0.45 μm). A Waters Delta 600 with 600 Controller and Waters 2996 Photodiode Array (Milford, MA, USA) equipped with an autosampler, online degasser and column heater was used to analyze the filtered sample. Data was evaluated and processed using the installed Millenium 32 Software (Waters Product). The filtered samples were separated at 27°C on a minibore Phenomenex Luna 5 μm C_18_ column (dimensions 250 × 4.60 mm) using a one-step linear gradient. The solvents were (A) 0.1% aqueous formic acid and (B) acetonitrile and the elution system was as follows: Initial conditions were 85% A and 15% B with a linear gradient reaching 25% B at t = 12 minutes. This was maintained for 10 minutes after which the programme returned to the initial solvent composition at t = 25 minutes and continued for 10 minutes. The flow rate used was 1 ml/min and the injection volume was 10 μl. The chromatogram was monitored at 254 and 366 nm. In addition, the extract was also spiked with pure flavonoids, namely rutin and quercitrin, and the chromatograms obtained were compared to the chromatograms of the pure standards of flavonoids, respectively.

### Antioxidant activity of MEDL

#### Total phenolic content

Total phenolic contents (TPC) of all plant extracts were determined using Folin-Ciocalteu reagent as described by Singleton and Rossi [22] with slight modifications. Methanolic solution of the extract in the concentration of 1 mg/ml was used in the analysis. In brief, 1 mg MEDL was extracted with 1 ml 80% methanol containing 1% hydrochloric acid and 1% distilled water at room temperature on the shaker set at 200 rpm for 2 hours. Then, the mixture was centrifuged at 2817 × g for 15 minutes and supernatant decanted into vials. The reaction mixture was prepared by mixing 200 μl supernatant extract, 400 μl (0.1 ml/0.9 ml) Folin-Ciocalteu reagent and allowed to stand at room temperature for 5 minutes. Then, 400 μl sodium bicarbonate (60 mg/ml) solution was added and the mixture was allowed to stand at room temperature for 90 minutes. The absorbance was determined using a spectrophotometer at 725 nm. A calibration curve was generated by using the gallic acid standard optical density, and the levels in the samples were expressed in terms of gallic acid equivalent (GAE) - TPC mg/100. Based on the measured absorbance, the concentration of phenolics was read (mg/ml) from the calibration line.

#### DPPH radical scavenging activity

The ability of MEDL to induce free radicals scavenging action was determined using the 2, 2-diphenyl-1-picrylhydrazyl (DPPH) assay according to the method of Blois [23] but with modifications involving the use of a high-throughput microplate system. Forty microliters of extracts (1 mg/ml) were mixed with 50 μl DPPH (FG: 384.32) (1 mM in ethanolic solution) and 150 μl absolute ethanol on a 96-well microtiter plate in triplicate. The 96-well microtiter plate was shaken for 15 seconds at 500 rpm. Then, the plate was left to stand at room temperature for 30 minutes and the absorbance was recorded at 520 nm.

#### Superoxide anion radical scavenging

The ability of MEDL to induce the superoxide anion radicals scavenging action was measured according to the method described by Liu et al. [24] with slight modifications. Briefly, the superoxide radicals are produced in phenazine methosulphate - nicotinamide adenine dinucleotide (PMS – NADH) systems via oxidation of NADH and evaluated by the reduction of nitroblue tetrazolium (NBT). In these experiments, the superoxide radicals were produced in 3 ml of Tris-HCl buffer (16 mM, pH 8) containing 1 ml of NBT (50 μM), 1 ml NADH (78 μM) and MEDL (25 – 50 μg). The reaction was started by adding 1 ml of PMS solution (10 μM) to the mixture. The reaction mixture was incubated at 25°C for 5 minutes, the absorbance was read at 560 nm using a spectrophotometer (Schimadzu UV-Vis 1700) against blank samples using l-ascorbic acid as a control. Decreased absorbance of the reaction mixture indicated increasing superoxide anion scavenging activity. The percentage inhibition of superoxide anion production was evaluated using the following formula:

$$\%\;\mathrm{inhibition}=\left[\left(A_{C}-A_{T}\right)/A_{C}\right]\;\times \;100$$

where A_C_ was the absorbance of the control (l- ascorbic acid), and A_T_ was the absorbance in the presence of MEDL or standards.

#### Oxygen radical absorbance capacity

The antioxidant capacity of MEDL was also determined using the oxygen-radical absorbance-capacity (ORAC) assay as described by Huang et al. [25], with some modifications. In the assay, 2, 2-azobis (2-amidinopropane) dihydrochloride (AAPH) was dissolved in 10 ml of 75 mM phosphate buffer (pH 7.4) which was prepared daily as peroxyl-radical generator. A fluorescein stock solution (1 mM) was prepared in 75 mM phosphate buffer (pH 7.4) and stored in wrapping foil at 5°C. During the analysis, the sodium fluorescein stock solution was diluted 1: 100 000 with 75 mM phosphate buffer (pH 7.4). The microplate wells were filled with 150 μl of working solution of sodium fluorescein and 25 μl of Trolox dilution in blank wells, or with 25 μl of MEDL extracts in sample wells and then was equilibrate by incubating for 10 minutes at 37°C. After the solutions equilibrated, 25 μl of 240 m M AAPH solution was added to the wells to initiate the reactions. BMG Omega Fluostar Fluorescent Spectrophotometer (Company, state & country) with injector was used with an excitation filter of 485 nm and an emission filter of 520 nm. The fluorescence intensity of each well was then measured kinetically with data taken every one minute for 3 hours. ORAC values were calculated using MARS Data Analysis Reduction Software.

#### Animals

Male Sprague Dawley rats (180-200 g; 8-10 weeks old) and male ICR mice (25-30 g; 5-7 weeks old) were obtained from the Veterinary Animal Unit, Faculty of Veterinary Medicine, Universiti Putra Malaysia (UPM), Malaysia and were housed at room temperature (27 ± 2°C; 70 – 80% humidity; 12 h light/darkness cycle) in the Animal Holding Unit (UPM). They were supplied with food and water *ad libitum* from the beginning of the experiments. The rats were handled in accordance with current UPM guidelines for the care of laboratory animals and the ethical guidelines for investigations of experimental pain in conscious animals. All experiments were conducted between 09.30 and 18.30 h to minimize the effects of environmental changes. The study protocol of the present study was approved by the Animal House and Use Committee, Faculty of Medicine and Health Sciences, UPM (Ethical approval no.: UPM/FPSK/PADS/BR-UUH/00449).

### Hepatoprotective assay

#### Carbon tetrachloride-induced hepatotoxicity test

For this *in vivo* study, male Sprague-Dawley rats weighing 180–200 g were used. The animals were kept in separate cages with *ad libitum* access to food and water in a room with controlled temperature (22 ± 3°C) and on a 12-hour light/dark cycle with lights switched on at 7:00 a.m. The animals were divided into 6 groups comprising 6 rats in each group as described below:

• Group I: only 10% DMSO orally (p.o.) for 7 days + 50% olive oil on day 7

• Group II: 10% DMSO p.o. for 7 days + CCl_4_ on day 7

• Group III: 200 mg/kg silymarin p.o. for 7 days + CCl_4_ on day 7

• Group IV, V, and VI: 50, 250, and 500 mg/kg of MEDL p.o. for 7 days + CCl_4_ on day 7

Each group received respective dose of the solution and extract once daily for 7 consecutive days. We administered 1 ml/kg of 50% CCl_4_ on day 7 to all animals except Group 1 and animals were sacrificed by exposure to diethyl ether 48 hours after administration of CCl_4_. Three millilitres of blood were collected by cardiac puncture. Blood samples were collected into lithium heparinized tubes using a sterile disposable syringe for biochemical analysis. After 20 minutes, the blood was separated by centrifugation for 10 minutes at 1000 × g using a refrigerated centrifuge. Following centrifugation, the plasma was transferred into a clean polypropylene tube and stored at -80°C. Liver was removed from rats and weighed.

### Biochemical analysis

Biochemical parameters were assayed according to standard methods. The enzymes alanine aminotransferase (ALT), alkaline phosphatase (ALP), and aspartate aminotransferase (AST) were measured using a Hitachi 902 Automatic Chemical Analyser.

### Histopathology

After the liver tissue was fixed in 10% formalin, specimens were embedded in paraffin, sectioned (3–5 μm), and stained with hematoxylin and eosin. The histochemical sections were evaluated under an electron microscope and evaluated by a pathologist according to the severity of the hepatic injury as described by El-Beshbishy et al. [26] with modifications.

### Statistical analysis

All the data are presented as mean ± SEM. Statistical analysis was performed using GraphPad Prism version 5. Data obtained were analyzed using the one-way analysis of variance and the differences between groups and the control group were determined using Dunnett’s post hoc test with P < 0.05 as the limit of significance.

## Results

### Phytochemical screening of MEDL

Phytochemicals screening of *D. linearis* was carried out using two types of samples, which are the powdered leaf and MEDL. From the results obtained, several phytoconstituents were detected. Only saponins, flavonoids, tannins and polyphenolic compounds, steroids, but not alkaloids, were detected in the respective leaves and MEDL. In comparison to MEDL, the leaves of *D. linearis* show no presence of triterpenes at all.

### HPLC analysis of MEDL

**Figure 1:**
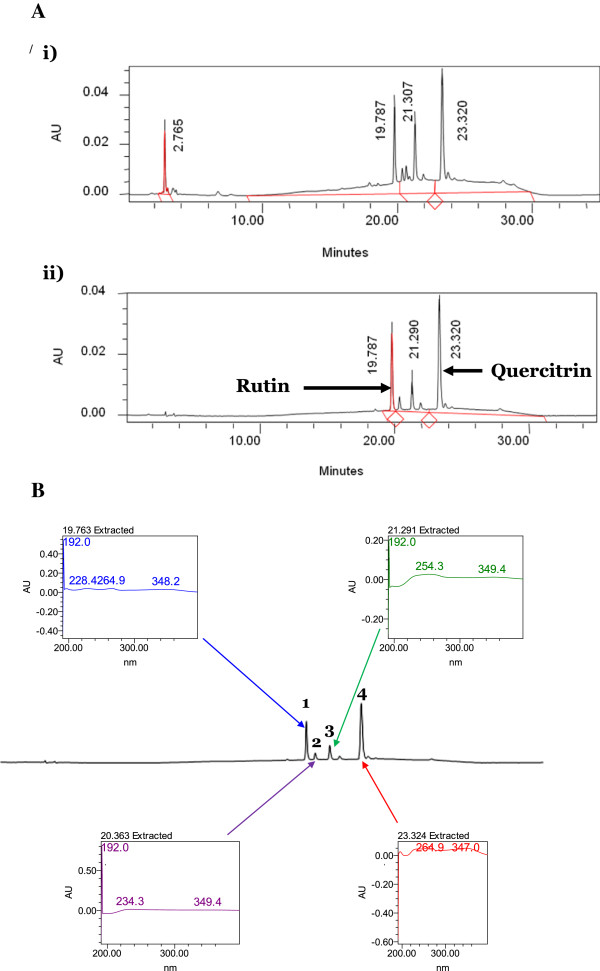
**The HPLC analysis of MEDL. A**. HPLC profile of MEDL at two different wavelength; i) 254 nm, and ii) 366 nm. Attempt was made to standardize the MEDL using various pure flavonoids standard. Based on the HPLC analysis, rutin and quercitrin was detected in the extract. **B**. HPLC profile of MEDL at 366 nm shows four major peaks, labelled as 1, 2, 3 and 4, that was successfully separated. Each peak was represented by their respective UV-Vis spectra with λ_max_ value of 228-349, 234-350, 254-350 and 264-347 nm, respectively. These ranges of UV-Vis spectra indicated the presence of flavonoids-types of compounds.

In an attempt to establish the HPLC profile of MEDL, the extract underwent the HPLC analysis at several wavelengths of which the peaks were clearly separated at the wavelengths of 254 and 366 nm (Figure 1A). At 366 nm, in particular, four major peaks were clearly detected in the chromatogram at the respective retention time (R_T_) of 19.76, 20.36, 21.29, and 23.32 minutes, respectively. Further analysis demonstrated that the four peaks showed λ_max_ values in the region of 228-349, 234-350, 254-350 and 264-347 nm, respectively (Figure 1B). The extract spiked with either rutin or quercitrin was found to share the same peak, respectively, indicating their presence in the MEDL (Figure 1A).

### Antioxidant activity of MEDL

For total phenolic content, the absorbance values resulted from the reaction between the extract solution and Folin-Ciocalteu reagent was compared to the standard solutions of GAE; the results of the colorimetric analysis of the TPC of the MEDL was 1757.25 ± 29.39 mg GAEs/g extract. According to the standard procedure, any extract with the TPC value of >1000 mg GAEs/g extract is considered to have high TPC value. At the concentration of 200 μg/ml, the MEDL caused 98.94 ± 1.14% antioxidant activities against DPPH free radical as compared to the standard drug, 200 μg/ml ascorbic acid. For the superoxide anion radical scavenging, at the concentration of 200 μg/ml, the MEDL caused 93.2 ± 1.81% antioxidant activities against superoxide anion free radical as compared to the standard drug, 200 μg/ml ascorbic acid. The ORAC value for 200 μg/ml MEDL, which is expressed as μM Trolox Equivalent (TE)/100 g, was 24272.50 ± 2056.53.

### In vivo hepatoprotective study

#### Effect of MEDL on the body weight and liver weight after induction with CCl_4_

The body and liver weights of the rats after treatments are shown in Table 1. Overall, there were no significant differences of body weight and liver weight between the different experimental groups compared to the negative control group. However, the rats in the CCl_4_-intoxicated group exhibited significantly (P < 0.05) higher liver/body weight ratio when compared to rats in the vehicle control group. The administration of MEDL lowered the liver/body weight ratio and this was comparable to the effects observed in silymarin-treated rats.

**Table 1:** **Effect of MEDL on percentage change of body and liver weight in CCl**_**4**_**- treated rats**

**Treatment**	**Dose (mg/kg)**	**Body weight, BW (g)**	**Liver weight, LW (g)**	**LW/BW (%)**
Vehicle	-	208.7 ± 5.546	5. 852 ± 0.2873	2.797 ± 0.0726
Vehicle + CCl_4_	-	208.9 ± 6.539	10.34 ± 0.2623^a^	4. 969 ± 0.1627^a^
Silymarin + CCl_4_	200	222.1 ± 4.42	7.830 ± 0.5425^b^	3.523 ± 0.2324^b^
MEDL + CCl_4_	50	204.6 ± 1.893	8.672 ± 0.2498^b^	4.237 ± 0.1902^b^
	250	220.4 ± 5.541	9.335 ± 0.2631^b^	4.240 ± 0.1020^b^
	500	209.3 ± 3.336	8.759 ± 0.4539^b^	4.181 ± 0.1924^b^

Values are expressed as means ± S.E.M. of six replicates.

^a^Significant different as compared to the normal control group in the respective column, p < 0.05.

^b^Significant different as compared to negative control group in the respective column, p < 0.05.

#### Histopathological study of the CCl_4_-induced hepatotoxic liver with and without pretreatment with MEDL

**Figure 2:**
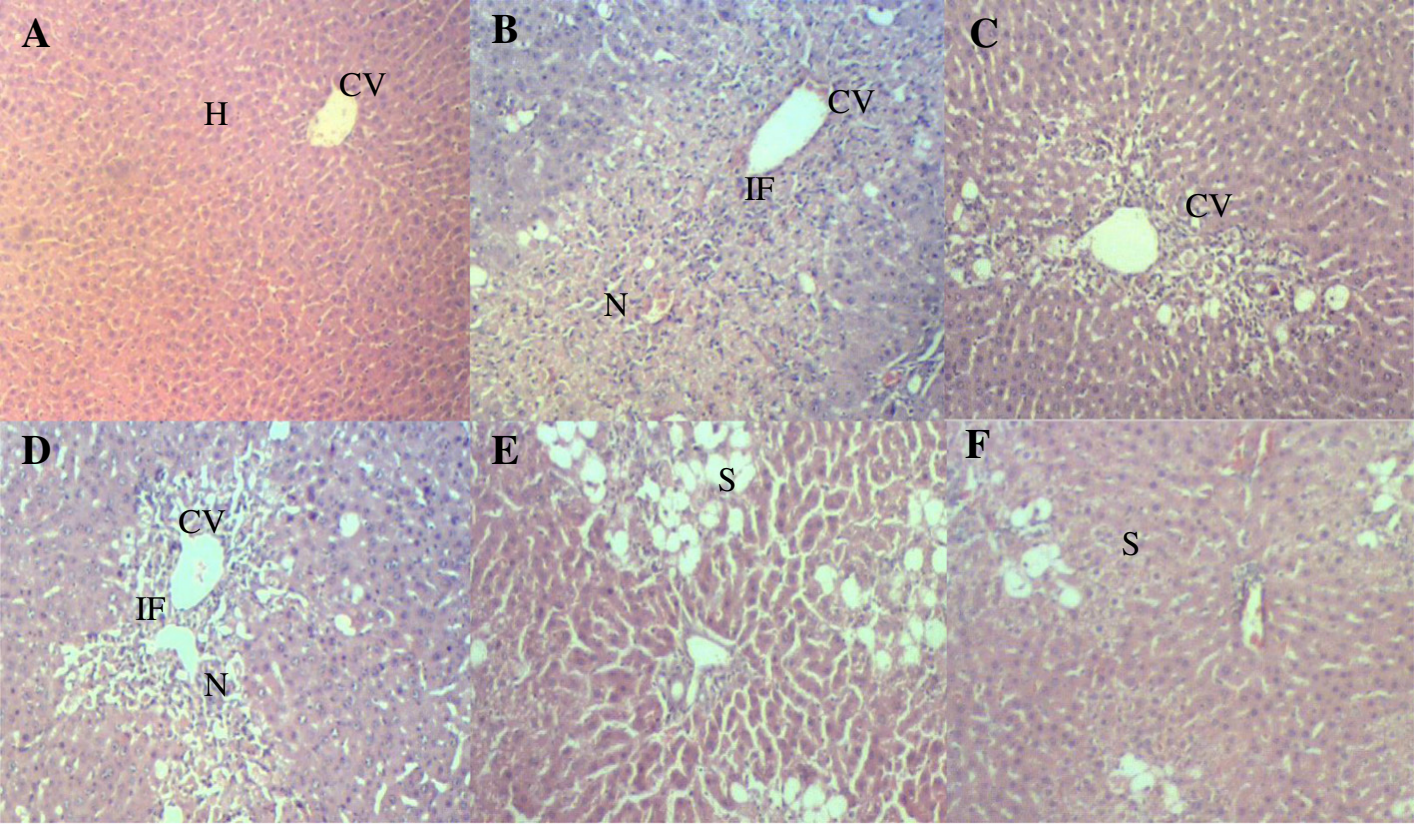
**Liver photomicrographs. A) Vehicle control** showed normal architecture of normal liver histology, central vein (CV), hepatocytes (H). **B)** Hepatotoxic liver after treatment with CCl_4_ showing ere necrosis (N) of the hepatocytes in parenchyma region, as well as macrovesicular steatosis (S). **C)** Pre-treatment with 200 mg/kg of Silymarin, positive control preserves normal architecture of the hepatocytes. **D)** Pre-treatment with 50 mg/kg of MEDL showing mild necrosis with mild infiltration of inflammatory cell **E)** Pre-treatment with 250 mg/kg of MEDL showing mild macrovesicular steatosis and absent to few degree of hepatocyte necrosis. **F)** Pre-treatment with 500 mg/kg of MEDL showing macrovesicular steatosis around central vein region. H & E staining, 40 × magnifications.

The livers of the normal control rats pretreated with 10% DMSO followed by treatment with 10% DMSO showed normal histoarchitecture. As seen in Figure 2A, the liver section of normal rat showed distinct hepatic cells with well-preserved cytoplasm, prominent nucleus, hepatocytes radiately arranged around the central vein, and well-defined sinusoidal line. The liver section of rats pretreated with 10% DMSO followed by treatment with CCl_4_ showed severe cytoplasmic vacuolation, microvesicular and macrovesicular fatty changes, karyopiknosis, loss of cellular boundaries, infiltration of inflammatory cells around the central vein and in the portal areas, congestion in the sinusoids, and necrosis of the liver cells (Figure 2B). Interestingly, those observations were markedly ameliorated in the groups pretreated with silymarin or MEDL indicated by significant reduction in the number of ballooning-degenerated hepatocytes and significant decreased in the necrosis area (Figure 2D-2F). In the group pretreated with 200 mg/kg silymarin followed by CCl_4_ administration, only slight steatosis and infiltration of lymphocytes and, spotty and focal necrosis were observed (Figure 2C). Pretreatment with MEDL exhibited a dose-dependent hepatoprotective effect wherein the group pretreated with 50 mg/kg MEDL exerted moderate necrosis and infiltration of leucocytes with mild steatosis. In the liver section pretreated with 250 mg/kg MEDL a moderate damage to the liver’s architecture was seen indicated by the presence of moderate steatosis with some fatty changes and mild leucocytes infiltration around the centrilobular region (Figure 2E). Figure 2F presents liver section of the group pretreated with 500 mg/kg MEDL showing slight necrosis and mild steatosis (Figure 2F). The histopatholgical scoring of CCl_4_-treated rat’s liver sections after pre-treatment with MEDL is shown in Table 2. The extract as well as silymarin caused remarkable decrease in the signs of CCl_4_-induced toxicity.

**Table 2:** **Histopathological scoring of the liver section of CCl**_**4**_**-induced hepatic injury rats pretreated with MEDL**

**Treatment**	**Dose (mg/kg)**	**Steatosis**	**Necrosis**	**Inflammation**	**Haemorrhage**
Vehicle + Vehicle	-	-	-	-	-
Vehicle + CCl_4_		++	+++	++	+
Silymarin + CCl_4_	200	+	+	+	-
MEDL + CCl_4_	50	++	+	++	-
	250	++	+	+	-
	500	+	+	+	-

The severity of various features of hepatic injury was evaluated based on those following scoring scheme: - normal, + mild effect, ++ moderate effect, +++ severe effect.

#### Biochemical analysis of blood samples

The changes in serum liver biomarkers are shown in Table 3. As indicated from the results, CCl_4_-intoxicated rats showed a significant (p < 0.05) increased in the activities of ALT, AST, and ALP compared to the normal control group. Pretreatment of rats with high dose of MEDL or silymarin significantly (p < 0.05) decreased the level of serum marker enzymes compared to the CCl_4_-intoxicated group.

**Table 3:** **Effects of MEDL on serum liver biomarkers of CCl**_**4**_**- treated rats**

**Treatment**	**Dose (mg/kg)**	**ALT (U/L)**	**AST (U/L)**	**ALP (U/L)**
Vehicle	-	16.00 ± 2.867	93.18 ± 6.239	114.7 ± 7.055
Vehicle + CCl_4_	-	4291 ± 665.3^a^	3456 ± 633.2^a^	460.0 ± 49.62^a^
Silymarin + CCl_4_	200	249.1 ± 30.68^b^	288.1 ± 25.32^b^	190.2 ± 19.67^b^
MEDL + CCl_4_	50	3283 ± 686.5	4291 ± 592.0	350.2 ± 36.80 ^b^
	250	1886 ± 134.2^b^	1853 ± 86.77^b^	171.8 ± 13.04^b^
	500	2298. ± 359.9^b^	1850 ± 228.5^b^	185.8 ± 10.23^b^

Values are expressed as means ± S.E.M. of six replicates.

^a^Significant different as compared to the normal control group in the respective column, p < 0.05.

^b^Significant different as compared to negative control group in the respective column, p < 0.05.

## Discussion

CCl_4_ has been widely used in animal models to investigate chemical-induced liver injury. The CCl_4_-induced experimental damage involves the formation of free radicals and the occurrence of lipid peroxidation in cellular and organelle membranes [27]. CCl_4_ will be metabolized in the liver to form highly reactive trichloromethyl free radicals (·CCl_3_), which are responsible in triggering the toxicity processes in the liver. This free radicals are further converted to trichloromethyl peroxyl radical (CCl_3_OO·) that act as the initiator of lipid peroxidation [28-30]. The presence of excessive amount of free radicals accelerate peroxidative degradation of cellular membrane, which resulted in the breakdown of cell integrity and the leakages of ALT and AST, and, subsequently, lead to the elevation of serum ALT and AST levels. Moreover, several remarkable pathological characteristics associated with CCl_4_-induced hepatotoxicity, namely fatty liver, cirrhosis and necrosis, could be seen and have been thought to result from the formation ad action of reactive intermediates (i.e trichlorometyl free radicals (CCl_3+_) metabolized by the mixed function cytochrome P450 in the endoplasmic reticulum.

One of the ways to measure the extent of hepatic damage is through the determination of the level of cytoplasmic enzymes (i.e. ALT, AST and ALP) that leak into the blood circulation, which is associated with massive centrilobular necrosis, ballooning degeneration and cellular infiltration of the liver [31]. Measurement of ALT is more liver-specific to determine hepatocellular damage [32]. However, measurement of AST is still commonly used to assess liver function because it is a sensitive indicator of mitochondrial damage, particularly in centrilobular regions of the liver [33]. According to Ahmed and Khater [34], transaminase levels return to normal due to the healing of hepatic parenchyma and the regeneration of hepatocytes. In the present study, CCl_4_–induced increase in the liver/body weight ratio indicating that the liver damage model had been successfully developed. This is further supported by the increase in the level of serum ALT, AST and ALP and histological scoring and microscopic findings. Interestingly, pretreatment with MEDL effectively protected the rodents against CCl_4_-induced hepatic intoxication, which are evidenced by significant reduction in the liver/body weight ratio, levels of serum liver enzymes. Furthermore, it is well established that intoxication with CCl_4_ leads to extensive necrosis in the liver centrilobular regions around the central veins [9] and fatty infiltration [35]. Interestingly, the microscopic examination also revealed the ability of pre-treated MEDL to reduce inflammation, steatosis and necrosis as indicated by decreased in histological scoring.

As described earlier, the basis of CCl_4_-induced liver toxicity lies in its biotransformation to free radicals via the cytochrome P450 system [36]. Since free radicals play important role in CCl_4_-induced hepatotoxicity, it seems rational to suggest that compounds with capability to neutralize such free radicals might also possess a hepatoprotective activity. In fact, various natural products (e.g extract of *Artemisia campestris* and pure compound like ginsenosides) with antioxidant potential have been reported to protect against CCl_4_-induced hepatotoxicity [36]. In the present study, MEDL was proven to exhibit radicals scavenging activities using various *in vitro* antioxidant models suggesting that the extract perhaps protected the hepatocytes by ameliorating oxidative stress and inhibiting lipid peroxidation. In addition, it is also plausible to add anti-inflammatory activity as another possible mechanism through which MEDL exerts the hepatoprotective activity. This suggestion is based on previous reports that *D. linearis* possess anti-inflammatory activity, which might explained the ability of MEDL to reduce inflammation associated with CCl_4_ as observed during microscopic examination.

MEDL has also been shown to contain high TPC value, which have been associated with high antioxidant activity [37,38] as well as anti-inflammatory activity [39,40]. These claims are in line with previous findings by Wu et al. [27] who reported that TPC-rich extract of *Laggera pterodonta* exerts both hepatoprotective and antioxidant activities when assessed using the *in vitro* primary cultured neonatal rat hepatocytes. Adetutu and Owoade [41] also reported that TPC-rich extract of *Hisbiscus sabdariffa* exhibits both hepatoprotective and antioxidant activities when assessed using *in vivo* CCl_4_-induced rat hepatotoxic model. The positive correlations between the high antioxidant activity and high TPC have been reported elsewhere [27,41] and are parallel with our findings. Thus, the antioxidant activity attributed by the presence of high concentration of TPC in MEDL could be suggested to play significant role in the observed hepatoprotective activity. Interestingly, one class of compounds that contributed to the high TPC reading is flavonoids, which have been known to play remarkable role in anti-inflammatory activity. Flavonoids have been detected in MEDL whereas MEDL has been shown to attenuate inflammation in CCl_4_-induced liver injury. This seems to suggest the role of anti-inflammatory activity of *D. linearis* as reported previously in enhancing the observed hepatoprotective activity.

Phytochemical analysis of MEDL demonstrated the presence of several phytocontituents (e.g. flavonoids, tannins, saponins and triterpenes) essentially associated with antioxidant and/or hepatoprotective effects. Earlier studies have claimed that phenolic compounds possess diverse pharmacological effects (i.e. antioxidant, anti-inflammatory, and hepatoprotective) [27,30,42]. MEDL exerted prominent hepatoprotective effects against CCl_4_-induced liver damage possibly via the antioxidant- and anti-inflammatory-modulated mechanisms, which is in compliance with the description on the pharmacological properties of phenolic compounds in the aforementioned reports. HPLC analysis of MEDL demonstrated the presence of four major peaks with their λ_max_ value that fall in the region of 192-348.2, 192-349.4, 192-349.4, and 264.9-347 nm, respectively. Based on report made by Tsimogiannis et al. (2007), each of the peaks represents flavonoid-based compounds. According to Tsimogiannis et al. (2007), flavonoids are divided into several subgroups, namely flavonols, flavones, dihydroflavonols, flavanonols and flavanones based on the UV-Vis spectra within which they fall. The UV-Vis spectra of flavonoids falls within two absorbance bands, labeled as Band A and Band B wherein Band A represents flavones or flavonols and lies in the range of 310–350 nm or 350–385 nm, respectively. Meanwhile Band B falls in the range of 250 and 290 nm and is much the same in all the aforementioned flavonoid subgroups. As for the flavanones and dihydroflavonols subgroup, Band A falls between 300–330 nm while Band B was detected in the range of 277–295 nm. Interestingly, the presence of low amount of flavonoids in MEDL is concurrent with the HPLC profile of MEDL that shows only four major peaks. Based on previous report, at least flavonoids such as rutin and quercitrin have been isolated from *D. linearis* and, interestingly, rutin and quercitrin have also been reported to exert antioxidant activity [43,44] and hepatoprotective activity [44,45]. Hence, flavonoids, like rutin and quercitrin, in addition to other polyphenolic constituents may be the prominent bioactive compounds responsible for antioxidant and hepatoprotective activities of MEDL.

It is also noteworthy to highlight on the inconsistency with regards to the presence of low amount of flavonoids in MEDL as detected in the phytochemical screening and the high TPC value of MEDL, and to provide possible explanation for this inconsistency. It is plausible to suggest that the high TPC value is attributed to the presence of other types of flavonoids, particularly, the flavonol 3-*O*- glycosides [46]. This suggestion, which is supported by the UV spectra characteristics of major peaks as detected in the HPLC, is based on the fact that flavonoid glycosides are soluble in methanol:water and acetone:water solvent systems, but not very soluble in water alone [47-49]. With the high presence of saponin in MEDL, the existence of flavonoids glycosides could be attributed to the tensioactive effect of saponins, which are present in relatively high quantities when compared to the flavonoids [50].

## Conclusions

The hepatoprotective potential of MEDL were evaluated for the first time. The results obtained demonstrated that MEDL possessed significant protection against CCl_4_-induced hepatotoxicity, which could be due, partly, to its high TPC value and, antioxidant and anti-inflammatory properties through scavenging free radicals to ameliorate oxidative stress and inhibit lipid peroxidation. Moreover, phytochemical screening revealed the presence of various phenolic compounds, with rutin and quercitrin identified, which may also contribute to the hepatoprotective activity of MEDL.

## Competing interest

The authors declare that there is no competing interest.

## Authors’ contribution

FHK, FY, SSM and MFFK carried out the animal studies, phytochemical screening, HPLC analysis, biochemical analysis and draft the manuscript. NM involved in the macroscopic and microscopic analysis and helped to draft the manuscript. LKT and MZS involved in the antioxidant study and statistical analysis. MNS participated in the design of the study and involved in the statistical analysis. ZAZ conceived of the study, participated in its design and helped to draft the manuscript. All authors read and approved the final manuscript.

## Pre-publication history

The pre-publication history for this paper can be accessed here:

http://www.biomedcentral.com/1472-6882/14/123/prepub
